# *Apocynum venetum*, a medicinal, economical and ecological plant: a review update

**DOI:** 10.7717/peerj.14966

**Published:** 2023-03-07

**Authors:** Tian Xiang, Longjiang Wu, Murtala Bindawa Isah, Chen Chen, Xiaoying Zhang

**Affiliations:** 1 Chinese-German Joint Laboratory for Natural Product Research, Qinba State Key Laboratory of Biological Resources and Ecological Environment, Shaanxi University of Technology, Hanzhong, Shaanxi, China; 2 Department of Biochemistry, Faculty of Natural and Applied Sciences, Umaru Musa Yar’adua University Katsina, Katsina, Nigeria; 3 Biomedical Research and Training Centre, Yobe State University, Damaturu, Nigeria; 4 Centre of Molecular and Environmental Biology (CBMA), Department of Biology, Campus de Gualtar, University of Minho, Braga, Portugal; 5 Department of Biomedical Sciences, Ontario Veterinary College, University of Guelph, Guelph, ON, Canada

**Keywords:** *Apocynum venetum* L, Medicinal plant, Fibrous plant, CiteSpace

## Abstract

*Apocynum venetum* L. is an important medicinal perennial rhizome plant with good ecological and economic value. Its leaves have many pharmacological effects such as anti-inflammatory, anti-depression, anti-anxiolytic, etc., while its fibers have the title of “king of wild fibers”. Furthermore, it was suitable for the restoration of degraded saline soil in arid areas. An increasing studies have been published in the past years. A scientometric analysis was used to analyze the publications of *Apocynum venetum* L. to clearly review the pharmacology, fiber application of *Apocynum venetum* L. and the potential value with its similar species (*Apocynum pictum Schrenk*) to the environment.

## Introduction

**Figure 1:**
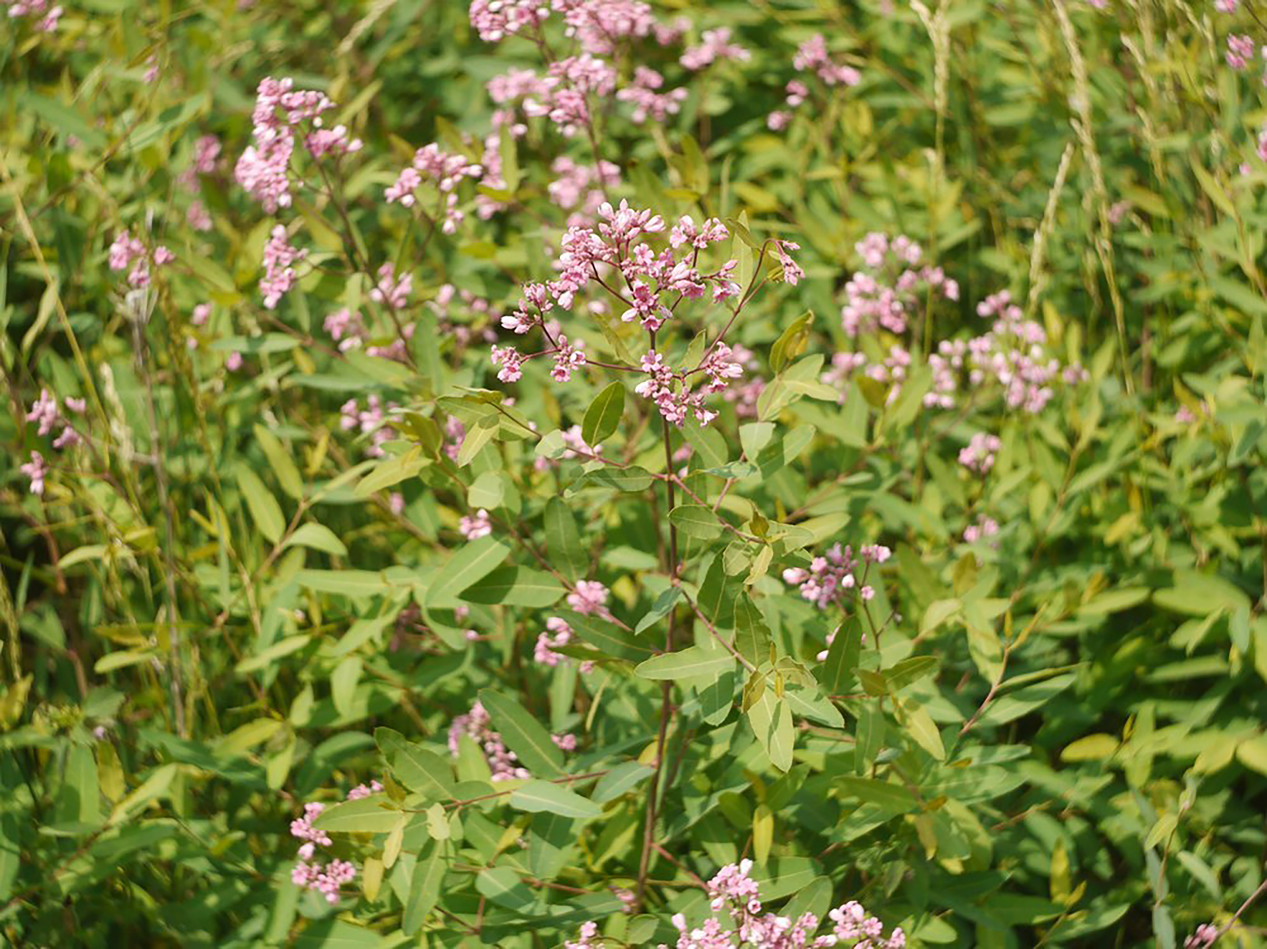
*Apocynum venetum* ssp. *Tauricum*. Image credit: Roman, https://www.inaturalist.org/photos/21168806.

*Apocynum venetum* L. (*A. venetum*), commonly known as “Luobuma” in Chinese and “Rafuma” in Japanese is a perennial herbaceous shrub (Fig. 1) widely distributed in the temperate regions of Asia, Europe and North America, especially in saline-alkali land, river-banks, fluvial plains and sandy soils (Grundmann et al., 2007; Jiang et al., 2021b; Xie et al., 2012). The species *Apocynum venetum* L. (Apocynaceae) currently includes 9 subspecies documented on World Flora Plant List (Table S1) (World Flora Online, 2022) *A. venetum* can adapt to extreme conditions where the surface salinity is up to 20% and the annual average precipitation is more than 250 mm, making the plant of high ecological value for the transformation of coastal saline and barren lands (Thevs et al., 2012; Yuan, Li & Jia, 2020a). *A. venetum* leaves has been used to produce herbal drugs and tea (Chinese Pharmacopoeia, 2020). Furthermore, since 2002 luobuma tea has been included in the list of health-care food in China (National Health Commission of the People’s Republic China, 2002). Lau et al. (2012) confirmed that *A.venetum* leaf extract could stimulate vascular receptor (alpha-adrenergic and angiotensin II receptors) and inhibit vasoconstriction, suggesting antihypertensive properties of the plant. Modern pharmacological investigations confirmed that *A. venetum* has, among other effects, anti-inflammatory, anti-depression, anti-anxiolytic, anti-ageing, antioxidants, cardiotonic and hepatoprotective effects (Du et al., 2020; Grundmann et al., 2007; Xie et al., 2015; Xie et al., 2012). *A. venetum* fiber, known as the “king of wild fibers”, is receiving increasing attentions in the apparel industry owing to its additional advantage of possessing antibacterial properties (Han et al., 2008; Wang, Han & Zhang, 2007; Xu et al., 2020a).

Alongside the rapid increase in *A. venetum*-related studies, systematic and comprehensive analyses on *A. venetum* publications is essential. We have previously reviewed the traditional uses, phytochemistry and pharmacology of *A. venetum* (Xie et al., 2012). As a timely update, this article aims to respond to the rapidly increasing literature on *A. venetum* studies by: (i) conducting scientometric analysis of the publications on *A. venetum* and (ii) reviewing the progress recorded on the exploration of the medicinal, economical and ecological benefits of the plant from 2012 to date. For the scientometric analysis, we used Citespace, which is a specifically designed to facilitate the detection of emerging trends and mutations in the scientific literature (Chen et al., 2012). Web of Science Core Collection (WoSCC; Clarivate Analytics, London, UYK) is the premier resource on the Web of Science platform. It is considered as the most trusted citation index on many research topics (Wu, Yakhkeshi & Zhang, 2022). This work can provide researchers and readers with a comprehensive information on *A. venetum*, covering the areas of phytopharmacy and pharmacology, functional food, ecology, and applications in textile and fiber industry.

## Survey Methodology

Data were collected from WoSCC with the following search strategy: Topical Subject = (“*Apocynum venetum*” OR “Luobuma”) OR Title = (“*Apocynum venetum*” OR “Luobuma”) OR Abstract = (“*Apocynum venetum*” OR “Luobuma”). The searched time spans 1987–2022, the type of literature was article and review, and the language was English. Our search strategy did not limit the impact factor of journals and the affiliation of authors. A total of 200 publications were obtained, including 190 articles and 10 reviews, and their full record with the cited references was exported in plain text format. CiteSpace 6.1.3 was used to analyze keywords of the literatures, with the time partition set to 1987–2022, the time slice set to 1, the node types set to keyword, G-index set to 25, and the pathfinder, pruning sliced networks and pruning the merged network were used to trim the atlas. Based on the result of keywords analysis of CiteSpace, the chapter topics were divided into the pharmacological effects and related components of *A. venetum*, *A. venetum* fiber, other *Apocynum* species similar to *A. venetum*: *Apocynum pictum Schrenk*, and the ecological value of *A. venetum* and *A. pictum,* and the topics were discussed. The discussion on the bioactive components cover the period 2012–2022.

### Keywords analysis of CiteSpace

**Figure 2:**
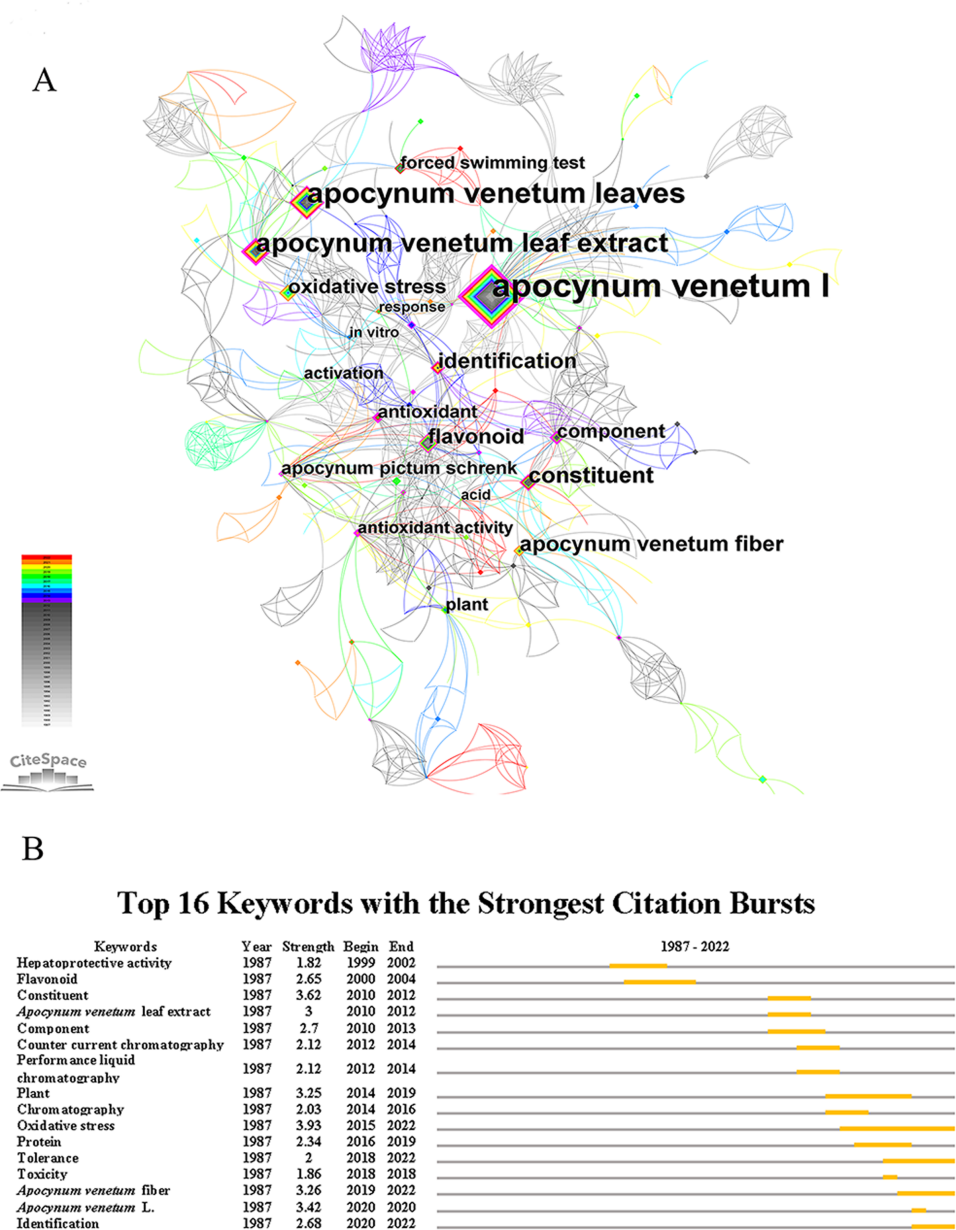
Keywords analysis of *A. venetum*. (A) Nodes in the network represent keywords. Node size represents the number of keyword occurrences. Node color: average time to appear, color from white to red, time from 1987 to 2022. (B) Top 16 keywords with the strongest citation bursts. The grey line represents time interval, the yellow line indicates time period in which a keyword was found to have a burst.

Keywords represent the core content of an article and provide information on the topic or the important category to which an article belongs. The keywords with high frequency and highly mediated centrality were analyzed and presented in the form of a visual mapping through the Citespace software (Fig. 2). The most frequent keywords from 1987–2022 were *Apocynum venetum* L. (111), *Apocynum venetum* leaves (52), *Apocynum venetum* leaf extract (31). The keywords with the highest centrality before 2018 include *Apocynum venetum* L. (0.49), component (0.28), hepatoprotective activity (0.27), identification (0.25) and antioxidant (0.23) (Fig. 2A, Table 1). However, after 2018, among the top ten keywords showing the highest centrality, two words that are poorly correlated with *Apocynum venetum* leaves appeared: *Apocynum venetum* fiber (0.28) and *Apocynum pictum* schrenk (0.27). These results implied that the studies before 2018 mainly focused on the components and the pharmacological effects of *Apocynum venetum* leaves while *Apocynum venetum* fiber and *Apocynum pictum Schrenk* have also attracted the attention of researchers in recent years.

**Table 1:** The top ten co-cited keywords with highest centrality.

Rank	Key words (1987–2018)	Counts	Centrality	Key words (2019–2022)	Counts	Centrality
1	*Apocynum venetum* l.	75	0.49	Antioxidant activity	4	0.77
2	Component	10	0.28	Acid	5	0.58
3	Hepatoprotective activity	3	0.27	Constituent	7	0.56
4	Identification	9	0.25	*Apocynum venetum* leaves	20	0.50
5	Antioxidant	6	0.23	Structural characterization	2	0.46
6	*Apocynum venetum* leaf extract	22	0.20	Oxidative stress	6	0.45
7	Mass spectrometry	2	0.20	Identification	9	0.30
8	*Apocynum venetum* leaves	37	0.18	*Apocynum venetum* l.	43	0.29
9	Apoptosis	3	0.18	*Apocynum venetum* fiber	10	0.28
10	Antioxidant activity	4	0.17	*Apocynum pictum* schrenk	3	0.27

Based on the keyword co-linear graph (Fig. 2A), the parameter of “burstiness” was set to *γ* = 0.5, minimum duration = 1. Sixteen burst entries were generated. Among them, the words that have kept the outbreak status were oxidative stress (3.93), *Apocynum venetum* fiber (3.26), identification (2.68) and tolerance (2.0) (Fig. 2B). These data confirmed that apart from the further in-depth pharmacological investigations, the fiber of this plant has recieved attention in recent years. In addition, the ecological value of *Apocynum venetum* L and *Apocynum pictum* Schrenk L has attracted increasing attentions.

### The bioactive components of *A.venetum*

#### Flavonoids

**Figure 3:**
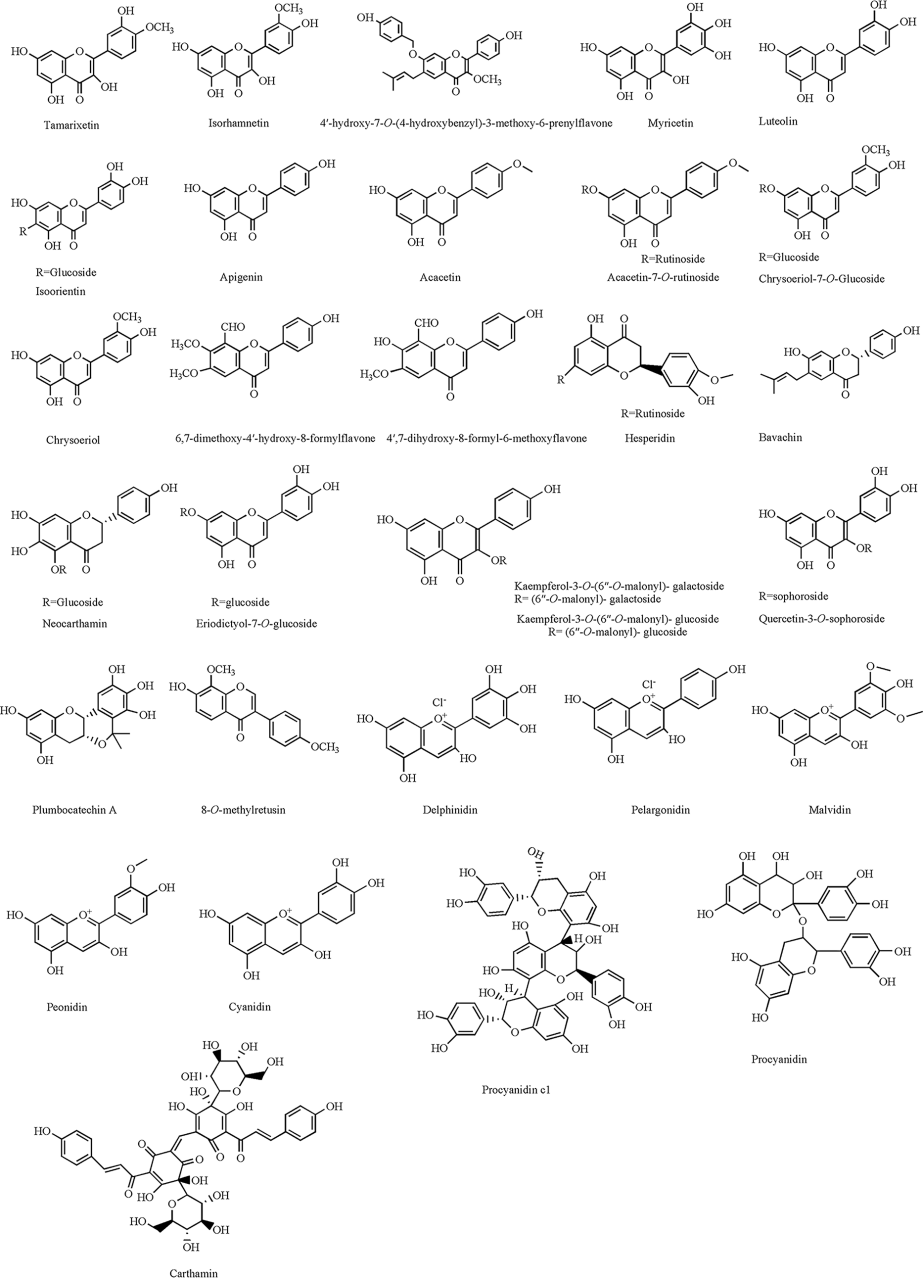
Chemical structures of the flavonoids isolated from *A. venetum* between 2012 to 2022.

With the deepening of research and the technological improvement in high performance liquid chromatography, mass spectrometry *etc*., many phytochemicals of *A.venetum* have been identified and isolated. Some of these phytochemicals were flavonoids such as hyperoside and isoquercetin, which bioactivities have been comprehensively reviewed previously (Xie et al., 2016a; Xie et al., 2016b; Xie et al., 2012). Since then, more studies have reported on the isolation and bioactivities of known and novel flavonoids from *A.venetum.* The flavonoids isolated from *A. venetum* since 2012 are listed in Table 2 and their structures shown in Fig. 3. Kaempferol, quercetin, isoquercitrin (quercetin-3-O-*β*-D-glucose) and astragalin (kaempferol-3-O-*β*-D-glucose) isolated from *A. venetum* leaves have significant anti-depressant activities in mice (Yan et al., 2016). Hyperoside isolated from the leaves of *A. venetum* showed antidepressant-like effect in P12 cell lines which could improve neuronal viability by protecting neurons from corticosterone damage (Zheng et al., 2012). Hyperoside had protective effect on H_2_O_2_-induced apoptosis of human umbilical vein endothelial cells (Hao et al., 2016). For acetaminophen-induced liver injury, both hyperoside and isoquercetin exerted hepatoprotective effect by upregulating the expression and activity of detoxifying enzymes such as sulfotransferases (hyperoside could also increase activities of UDP-glucuronosyltransferase) in liver microsomes and inhibited the activity of cytochrome P450 2E1, accelerating the harmless metabolism of acetaminophen. Additionally, isoquercetin could significantly inhibit acetaminophen induced oxidative stress and nitrosative stress (Xie et al., 2016a; Xie et al., 2016b). Isoquercitrin, isolated from the *A. venetum* leaf aqueous extract exerted anti-obesity effect in high fat diet induced obese mice by inhibiting adenosine 5′-monophosphate-activated protein kinase (AMPK)/sterol regulatory-element binding protein (SREBP-1c) signaling pathway, glucose uptake, and glycolysis flux. C-1-tetrahydrofolate synthase, carbonyl reductase, and glutathione S-transferase P are potential target proteins of isoquercitrin (Manzoor et al., 2022). 8-*O*-methylretusin (Fig. 3) isolated from *A venetum* leaves showed antifouling activity (Kong et al., 2014). On the other hand, 4′,7-dihydroxy-8-formyl-6-methoxyflavone isolated from *A venetum* leaves showed high anti-inflammatory activity *via* significant inhibitory effect on the production of nitric oxide (NO) and tumor necrosis factor-*α* (TNF-*α*) (IC_50_ values were 9.0 ± 0.7 and 42.1 ± 0.8 µM, respectively) in lipopolysaccharide-induced mouse peritoneal macrophages (RAW 264.7) (Fu et al., 2022).

**Table 2:** Flavonoids isolated from *A. venetum* between 2012 to 2022.

Class	Compound identified	Bioactivity	Plant part isolated from	Reference
Flavonols	Tamarixetin		70% methanol extract in *A. venetum* leaves	Gao et al. (2019), Yan et al. (2016)
	Isorhamnetin		95% ethanol extract in *A. venetum* leaves	Huang et al. (2017)
	4′-hydroxy-7-*O*-(4-hydroxybenzyl)-3-methoxy-6-prenylflavone	anti-inflammatory	The ethyl acetate -soluble extract of the leaves of *A. venetum*	Fu et al. (2022)
	Myricetin		75% ethanol extract in *A. venetum* leaves	Zhang et al. (2022)
Flavones	Luteolin	antidepressant	70% methanol extract in *A. venetum* leaves	Gao et al. (2019)
	Isoorientin			Gao et al. (2019)
	Apigenin			Gao et al. (2019)
	Acacetin			Gao et al. (2019)
	Acacetin-7-*O*-rutinoside			Gao et al. (2019)
	Chrysoeriol-7-*O*-glucoside			Gao et al. (2019)
	Chrysoeriol	anti-inflammatory	The ethyl acetate -soluble extract of the leaves of *A. venetum.*	Fu et al. (2022)
	6,7-dimethoxy-4′-hydroxy-8-formylflavone	anti-inflammatory		Fu et al. (2022)
	4′,7-dihydroxy-8-formyl-6-methoxyflavone	anti-inflammatory		Fu et al. (2022)
Flavonones	Hesperidin		70% methanol extract in *A. venetum* leaves	Gao et al. (2019)
	Neocarthamin			Gao et al. (2019)
	Bavachin	anti-inflammatory	The ethyl acetate -soluble extract of the leaves of *A. venetum.*	Fu et al. (2022)
Flavonol glycosides	Kaempferol-3-*O*-(6″-*O*-malonyl)- galactoside		70% ethanol extract in *A. venetum* leaves	An et al. (2013)
	Kaempferol-3-*O*-(6″-*O*-malonyl)- glucoside			An et al. (2013)
	Eriodictyol-7-*O*-glucoside		70% ethanol extract in *A. venetum* leaves	Zhao et al. (2014)
	Quercetin-3-*O*-sophoroside		83% methanol extract in *A. venetum* leaves	Wang et al. (2020)
Flavan-3-ols	Plumbocatechin A	radical-scavenging activity	The ethyl acetate fraction of the methanol extract	Kong et al. (2014)
Isoflavones	8-*O*-methylretusin	antifouling activities		Kong et al. (2014)
Anthocyanidins	Delphinidin		70% methanol extract in *A. venetum* leaves	Gao et al. (2019)
	Pelargonidin			Gao et al. (2019)
	Malvidin			Gao et al. (2019)
	Peonidin			Gao et al. (2019)
	Cyanidin			Gao et al. (2019)
Proanthocyanidins	Procyanidin c1			Gao et al. (2019)
	Procyanidin			Gao et al. (2019)
Chalcones	Carthamin			Gao et al. (2019)

Wang et al. (2020) investigated the absorption and metabolism of quercetin-3-*O*-sophoroside, isolated from the leaves of *A. venetum,* in rats. The results indicated that quercetin-3-*O*-sophoroside was completely absorbed in the small intestine and metabolized in the jejunum to sulfated quercetin-3-*O*-sophoroside, methylated quercetin-3-*O*-sophoroside, and methylated quercetin-3-*O*-sophoroside sulfate. Quercetin-3-*O*-sophoroside was deglycosylated to aglycones by the cecal microbiota to form derivatives of benzoic, phenylacetic and phenylpropionic acids (Wang et al., 2020).

To obtain larger amounts of flavonoids, *A. venetum* hairy roots were induced with *Agrobacterium rhizogenes* strain Ar.1193, and 117 kinds of flavonoids were detected in the roots. The flavonoid content and antioxidant activity of the roots were significantly increased as compared to field-planted roots, therefore, this technique could be used for large-scale production of flavonoids from *A. venetum* (Zhang et al., 2021).

#### Polysaccharides

Natural polysaccharides have been proved to possess, among other effects, immune regulatory, anti-oxidative and anti-inflammatory activities, as well as having the advantages of being safe and non-cytotoxic (Liu et al., 2022). Zhou et al. (2019) used different concentrations and kinds of solvents (HCl, H_2_O, NaOH) to extract polysaccharides from *A. venetum* leaves. The results showed that the polysaccharide yield was the highest with 21.32% (w/w), 0.5 M NaOH at 90 °C, and the bioactivity of the alkaline extracted polysaccharides was the strongest, which was reflected in the antioxidant capacity (DPPH and ABTS radical scavenging activities) and *α*-glucosidase and lipase inhibitory activities. The 0.5 M NaOH extracted polysaccharides showed a strong inhibitory activity on *α*-glucosidase (IC50 value of 16.75 µg/mL), which was better than the positive control, acarbose (IC50 value of 1,400 µg/mL). In addition, the alkaline polysaccharide-rich extracts were proved to possess hypoglycemic and hypolipidemic effects on mice with high fat diet induced and streptozotocin-induced type 2 diabetes. Moreover, the extract reversed intestinal dysbiosis by increasing the abundance of *Odoribacter*, *Anaeroplasma*, *Muribaculum*, *Parasutterella* and decreasing the abundance of *Enterococcus*, *Klebsiella*, *Aerococcus* in diabetic mice (Yuan et al., 2020b).

Some polysaccharides were also isolated from various parts of *A. venetum* and validated for bioactivity. These are summarized in Table 3. ALRPN-1 and ALRPN-2 exerted a significant anti-inflammatory activity in lipopolysaccharide-induced macrophages by regulating the levels of pro-inflammatory mediators (NO) and cytokines (TNF-*α*, interleukin-6, interleukin-1 *β*) and the mechanism may involve, in part, extracellular signal-related kinase (ERK)/mitogen-activated protein kinases (MAPKs) signaling pathway (Liu et al., 2022). Vp2a-II and Vp3 obtained from the flowers of *A. venetum* showed anticoagulant activity and immunoregulation. The anticoagulant activities of Vp2a-II and Vp3 were assayed *in vitro* by plasma coagulation parameters (activated partial thromboplastin time (APTT), thrombin time (TT), prothrombin time (PT), fibrinogen). The results showed that Vp3 significantly prolonged TT and PT, while Vp2a-II significantly prolonged APTT and TT, indicating that the two polysaccharides could inhibit blood coagulation (Wang et al., 2019b). In addition, the polysaccharides could exert immunomodulatory effects by promoting phagocytic activity, enhancing NO secretion and mRNA expression of inducible nitric oxide (iNO) synthase, interleukin-6 and TNF-*α* which activate RAW264.7 cells. Vp2a-II might activate the MAPK signaling pathway, which then induce the nuclear translocation of NF-*κ*B p65 (Wang et al., 2022).

**Table 3:** Polysaccharides from different parts of *A. venetum*.

Name	Average molecular weight	Monosaccharide	Bioactivity	Mechanism	Plant part	Reference
ALRPN-1	1. 542 ×10^4^ Da	Glucose	Anti-inflammatory	ALRPN-1 and ALRPN-2 exert significant anti-inflammatory activity in LPS-induced macrophages by regulating the levels of pro-inflammatory mediators (NO) and cytokines (TNF- *α*, IL-6, IL-1 *β*) and activating the ERK/MAPKs signaling pathway.	A. *venetum* root	Liu et al. (2022)
		Galactose				
		Arabinose				
ALRPN-2	5.105 × 10^3^ Da	Glucose				
		Galactose				
		Mannose				
Vp2a-II	7 ×10^3^ Da	–	Anticoagulant activity	Vp2a-II could inhibit blood coagulation through exogenous pathways and endogenous coagulation pathways.	A. *venetum* flower	Wang et al. (2022); Wang et al. (2019a); Wang et al. (2019b)
			Immunoregulatiory	Vp2a-II and Vp3 could activate RAW264.7 cells by promoting cell viability phagocytosis, and enhancing the NO secretion and mRNA expression of iNOS, IL-6 and TNF- *α*. Moreover, Vp2a-II and Vp3 could trigger the MAPK signaling pathway and then induce the nuclear translocation of NF- *κ*B p65.		
Vp3	9 × 10^3^ Da	–				
			Anticoagulant activity	Vp3 could inhibit blood coagulation mainly through exogenous pathways and coagulation pathways.		
ATPC-A mixture (the polysaccharide conjugates contained three components)	5.50 × 10^4^ Da 5.38 × 10^4^ Da 5.67 × 10^3^ Da	Mannose	Emulsifying properties	–	A. *venetum* tea (made of A.*venetum* leaves) residues	Chen et al. (2022a), Chen et al. (2022b)

In addition to the pharmacological effects of *A. venetum* polysaccharides, researchers have also began exploring their other properties. The polysaccharide conjugates (ATPC-A) extracted from *A. venetum* tea residues with an alkaline solution (0.10 M NaOH) had emulsifying properties and stabilized the emulsion which comprised of amphipathic polysaccharides covalently bound to proteins. The stability of the neat ATPC-A emulsions with a concentration equal to or greater than 1.00 weight % was higher than 5.00 weight % gum arabic during storage at different temperatures and pH values (Chen et al., 2022b).

### Other phytochemical components of *A. venetum*

Many studies have reported other phytochemicals from *A. venetum* leaf extracts and their pharmacological effects. The ethanol extract of *A. venetum* leaf possesses anti-cancer activity. A fraction separated from the extract could inhibit the proliferation of Human PCa cells tumor cells. Lupeol accounted for approximately one-fifth (19.3% w/w) of the components of the fraction and was implicated for the induced cytotoxicity against PCa cells. The fraction and lupeol elicited similar anti-proliferative mechanisms, involving: regulating apoptosis signal molecules (P53, cytochrome c, Bcl-2, and caspase 3 and 8), promoting G2/M arrest through impairing the DNA repair system *via* downregulating the expression of uracil-DNA glycosylase, as well as downregulating the expression of *β*-catenin (Huang et al., 2017). In preventing D-galactose-induced oxidative damage in mice, the polyphenol extract of *A. venetum* was superior to the antioxidant vitamin C (Guo et al., 2020). Within its safe concentration range (0–100 µg/ml), the polyphenol extract of *A. venetum* inhibited U87 glioma cell proliferation and caused cell apoptosis by affecting NF- *κ*B and genes of other relevant pathways (Zeng et al., 2019). Additionally, *A. venetum* leaf extract inhibited doxorubicin induced cardiotoxicity through (protein kinase B) Akt/(B-cell lymphoma-2) Bcl-2 signaling pathway (Zhang et al., 2022). The efficacy and mechanism of action of individual chemical components, as well as their possible synergistic effects, of *A. venetum* leaf extract need to be further investigated.

In addition to flavonoids, polysaccharides and polyphenols, sterols (*β*-sitosterol, sitgmasterol), triterpenoids (lupeol, uvaol), glycolipids (apocynoside I), natural lignan glycoside (alloside of benzyl alcohol) and amino acids have been isolated from *A. venetum* (Huang et al., 2017; Sun et al., 2022). *A. venetum* flowers are rich in free amino acids, accounting for about 3% of the total dried weight, including leucine (13.71 µg/mg), isoleucine (7.86 µg/mg), lysine (2.22 µg/mg), tryptophan (1.67 µg/mg) and valine (1.20 µg/mg) (Jin et al., 2019). Uvaol from *A. venetum* leaves had potent anti-inflammatory effects on dextran sulfate sodium-induced experimental colitis and lipopolysaccharide-stimulated RAW264 cells (Du et al., 2020). Validation of the activities of other components in *A. venetum* should be the focus of future studies.

### *A. venetum* fiber

The fiber of *A. venetum* has been used in textile and paper industries with superior properties compared to other commonly used fibers. Fiber from *Apocynum* species has a higher average length to diameter ratio (up to 1219) compared to kenaf (209), another natural plant fiber (Liu et al., 2020; Wang, Han & Zhang, 2007; Xie et al., 2012). Another reason for the popularity of *A. venetum* fabric is the antibacterial effect that *A. venetum* fiber naturally possesses (Li et al., 2012; Song et al., 2019). Such antibacterial activity might be because: (i) *A. venetum* fiber has small openings between microstructures, which improve the breathability of the *A. venetum* fabric, which subsequently destroy the environment for bacterial growth (Han et al., 2008); (ii) the *A. venetum* stem cells contain tanning agents, which is resistant to microbial decomposition (Thevs et al., 2012); (iii) the presence of water-insoluble polyphenol derivatives confers antimicrobial properties to the fabric (Xu et al., 2020a).

*A. venetum* is rich in cellulose, but impurities such as pectin, lignin, and waxes must be removed to produce clean fibers (Lou et al., 2019). In the direction of environmental safety and high efficiency, various degumming methods have been proposed, including chemical degumming, biological degumming and microwave-assisted ultrasonic degumming. A study revealed that microwave-assisted ultrasonic degumming showed the advantages of requiring less chemical reagents during degumming (1 kg raw *A. venetum* bast needed 0.6 kg of reagents while the chemical degumming treatment required 1.34 kg) and shorter time, as well as higher quality (low residual gum content of 5.15%; lignin content less than 3%; whiteness more than 80% in the refined *A. venetum* fibers) (Li et al., 2020). Degumming methods and the fiber quality of *A. venetum* reported from 2012 to 2022 are listed in Table 4.

**Table 4:** Degumming methods and the quality of fiber obtained from *A. venetum* (studies between 2012 to 2022).

Degumming type	Processing method	Fiber quality	Impact on the environment	Reference
Bio-chemical combined degumming process	*Apocynum* fibers > > Boiling (12 g/L pectinase, Material: Liquor (M: L)-1:30, time: 2 h, temperature: 50 °C, PH8-10) > > washing > > boiling (12 g/L NaOH, M: L-1:30, time: 1.5 h) > > washing > > bleaching (20 g/L H_2_O_2_, M: L-1:30, time: 1.5 h, temperature: 95 °C) > > washing > > oven-dried (temperature: 80 °C)	Fiber breaking strength: 22.84 cN/dtex; Whiteness: 73.9; Fineness:4.97 dtex; Crystallinity: 74.5%; Moisture regain: 7.7380%.	This method could reduce the pollution caused by chemicals.	Chen et al. (2022a), Chen et al. (2022b)
Biodegumming (Bacterial strain *Pectobacterium wasabiae*)	Oscillating fermentation (fermentation time: 12 h, inoculum size: 2%, M: L -1:10, temperature: 33 °C, shaking rate:180 rpm) > > boiling (temperature: 100 °C, time: 20 min) > > washing by machine	Residual gum content: 12.57%; Percentage of raw material weight loss: 30.05%; The fiber counts:1,002 m/g	Chemical Oxygen Demand: 3,119 mg/L	Duan et al. (2021)
Microwave-assisted ultrasonic degumming	Sample > > Microwave pretreatment (10 g/L NaOH, M: L-1:20, time: 20 min, temperature:120 °C, power: 600W) > > rinsing > > drying > > ultrasonic degumming > > soaking (10 g/L NaOH and 1 g/L H_2_O_2_, M: L-1:20, time: 60 min, temperature:50 °C, power: 800W, frequency: 28 Hz	Residual gum content: 5.15%; Fiber breaking strength: 7.67 cN/dtex; Fiber length:32.5mm; Whiteness: 83%; Fineness: 4.05 dtex;	For degumming 1 kg of raw AV bast needed 0.6 kg of chemical reagents	Li et al. (2020)
Chemical degumming	Stripped bast by machine > > pretreatment (0.2%Al_2_(SO_4_)_3_, room temperature, M: L- 1:15, time: 7h) > > fiber washing > > cooking (1%NaOH, 0.25% thiourea, M: L- 1:15, temperature:95 °C, time intervals:2, 3, 5 h) > > washing > > acid soaking (2% CH_3_COOH, room temperature, M: L- 1:15, time: 2 min) > > washing > > bleaching (2% H_2_O_2_, 0.1% tween-80 surfactant, temperature: 94 °C, M: L- 1:15, time: 1 h) > > washing > > drying (oven-dried at 105 °C).	Moisture regain: 7.0%; The cooking processes of three different time intervals: Residual gum content: 3.64, 3.03, 2.70%, respectively; Crystallinity: 81.14, 78.80 73.75%, respectively; Tenacity: 8.63, 7.00, 6.93 cN/dtex, respectively; Fiber diameter: 2.52, 2.37, 2.14 dtex, respectively.	The method uses metal salts of aluminum for pretreatment, which is more sustainable.	Halim et al. (2020)
Deep eutectic solvents (DES) with the assistance of microwave	DES Configuring (choline chloride and car bamide-1:2 molar ratio (w/w) > > oil bathing (temperature: 80 °C, M: L- 1:20, time: 1 h) > > immersing with microwave oven (temperature:110 °C, M: L- 1:20, time: 1 h ) > > washing > > cooking (1%NaOH, time: 1 h) > > washing > > oven-dried	Residual gum content: 6.54%; Fiber breaking strength:14.14 cN/dtex; Crystallinity: 77.92%. Average fiber fineness: 4.05 dtex.	DES reagent selected for this method is biodegradable	Song et al. (2019)
Degumming with Ionic Liquid (IL:1-butyl-3-methylimidazolium acetate-water mixtures.) Pretreatment	*A.venetum* fibers > > pretreatment > > water boiling (temperature: 70 °C, M: L- 1:20, time: 3 h) > > rinsing with hot water (60 °C) > > rinsing with tap water > > degumming with IL-water mixtures (80% IL-water mixtures, temperature: 90 °C M: L- 1:20, time: 4 h) > >chemical degumming (10 g/L NaOH and 2% Na_3_P_3_O_10_, M: L- 1:20 temperature: 95 °C, time: 2 h) > > acid rinsing (1.5 g/LH_2_SO_4_, room temperature, M: L- 1:20, time: 5 min) > > washing with tap water > > drying	Residual gum content: 3.90%; Fiber breaking strength: 452.7 cN/dtex; Fineness: 0.7 um Crystallinity:76.62%	Mild conditions and low toxicity.	Yang et al. (2019)
Chemical degumming	Pre-acid treatment (2% H_2_SO_4_, temperature: 60 °C, M: L- 1:15, time: 1 h) > > washing > > first-cooking (5% NaOH, 3% Na_2_SiO_3_, 2.5% Na_2_SO_3_, temperature: 100 °C, M: L- 1:10, time: 2.5 h) > > washing > > second-cooking (15% NaOH, 3% Na_2_SiO_3_, 2% sodium tripolyphosphate, temperature: 100 °C, M: L- 1:10, time: 2.5 h) > > washing > > acid rinsing (1 g/L H_2_SO_4_) > > washing > > dewatering > > shaking > > drying	Fiber breaking strength:401.56 cN/dtex; The average length:29.68 mm; Fineness:4673.25 nm; Color: reddish yellow; Moisture regain: 8.70%; Crystallinity:70.36%;	–	Lou et al. (2019)
Bio-degumming (*Pectobacterium* sp. DCE-01)	Machine rolling preprocessing > > bacteria culture (Pectobacterium sp. DCE-01, temperature: 34 °C, time: 6 h, speed: 180rpm, culture medium: 1.0% glucose, 0.5% NaCl, 0.5% beef extract, 0.5% peptone, and 100 mL water, pH 6.5–7.0.) > > Bacterial liquid preparation (water containing: 0.05% NH_4_H_2_PO_4_ and 0.05% K_2_HPO_4,_ pH 6.5–7.0) > > fermentation and degumming (temperature: 33 °C, M: L- 1:15, bacterial solution: fermentation water-2:100, time: 16 h, speed: 180 rpm) > > boiling (temperature: 33 °C, time: 20 min) > > washing by a fiber washer > > drying	Residual gum content: 12.22%; Fiber breaking strength: 5.47 cN/dtex;	Chemical Oxygen Demand: 3,245 mg/L	Duan et al. (2017)
A novel ionic liquid degumming	Boiling (1 g/L H_2_SO_4_, temperature: 50 °C, M: L- 1:20, time: 2 h ) > > washing (until the washings were neutral) > > degumming (80% 1-butyl-3-methylimidazolium acetate, temperature: 130 °C, M: L- 1:20, time: 3 h ) > > washing > > drying	Residual gum content: 9.80%; Fiber breaking strength: 4.64 cN/dtex; Length:24.44 mm Fineness: 4.10 dtex; Crystallinity:78.66%	The degumming process was mild compared to the traditional chemical process.	Yang et al. (2015)

In addition to the textile industry, *A. venetum* fiber also has many potential applications in medicine as well as in the construction industry. Microcrystalline cellulose (MCC-N) from *A. venetum* fibers was shown to have a rougher structure and less macrostructure than commercially available microcrystalline cellulose (MCC-C). MCC-N had a crystallinity of up to 78.63% and a thermal stability comparable to that of MCC-C, which made it suitable as a load-bearing material for composite structures, and could be used in polymer composites with high temperature resistance (Halim, 2021). Furthermore, cellulose nanofibers (CNFs) from *A. venetum* straw were added into poly lactic acid (PLA), and the prepared PLA/CNFs film did not only improve the wettability and permeability of PLA, but also had superior antibacterial properties (the antibacterial growth inhibition rate on *Escherichia coli* and *Staphylococcus aureus* were 96.31% and 92.83% at PLA/6% (w/w) CNFs film, respectively). Then, polyvinyl pyrrolidone was added to this film to form a sustained-release nanofiber membrane (PLA/drug-loaded PVP nanofiber membranes), and a purified sea buckthorn was embedded in the drug-loaded film to evaluate its performance. The nanofiber membrane extended and sustained the release of purified sea buckthorn, and the cumulative release reached a maximum of 75.41%. It showed the advantage of a profile with a high initial release followed by a slow diffusion phase (Wang et al., 2021b; Wang et al., 2019a). In addition, when the hydrogel was prepared with chitosan as the matrix, the addition of CNFs improved the mechanical properties and swelling rate of the chitosan-based hydrogel. As the CNFs was 1.5%, the compressive strength of the hydrogel increased by nearly 20%, the swelling capacity reached 140%. In this form, the antibacterial efficacy against *Escherichia coli* and *Staphylococcus aureus* were 98.54% and 96.15%, respectively (Wang et al., 2021a). See Abubakar, Gao & Zhu (2021) for further details on the composition, properties and degumming methods of *A. venetum* fiber.

### Other *Apocynum* species similar to *A. venetum*: *Apocynum pictum* Schrenk

Due to excessive exploitation, wild *A. venetum* has declined in recent years. A similar species, *Apocynum pictum* Schrenk (*Apocynum hendersonii* Hook) is often used in the market as a substitute for *A. venetum* due to their similarity in morphological characteristics and geographical distribution. The incorporation of *A. pictum* may affect the safety and effectiveness of *A. venetum* (An et al., 2013; Chan et al., 2015; Zheng et al., 2022). Although *A. pictum* has not been included in the Chinese Pharmacopoeia (Chinese Pharmacopoeia, 2020), some studies have reported that it is an important medicinal plant (Gao et al., 2021; Jiang et al., 2021a). For the quality control of *A. venetum* and to explore the potential application of *A. pictum*, some studies compared the similarities and differences between the two species in terms of genome size, flavonoid content, chemical composition and biological activity. The whole genomes of the two species were both small and similar, with 232.80 megabase (*A. venetum*) and 233.74 megabase (*A.pictum*). The contents of quercetin, hyperoside and total anthocyanin in *A. venetum* were much higher than those of *A. pictum*, which was considered to be the reason for the difference in color between the two species (Gao et al., 2019). Hyperoside could be a suitable chemical marker to distinguish between the two species (Gao et al., 2019). In addition, *A. venetum* has a better antioxidant activity than *A. pictum* (Chan et al., 2015). However, recent studies have shown that the flavonoids from *A. pictum* (quercetin-3-sophoroside, isoquercetin, quercetin-3-*O*-(6-*O*-malonyl)-galactoside) and *A. venetum* (hyperoside, isoquercetin, quercetin-3-*O*-(6-*O*-malonyl)-galactoside, quercetin-3-*O*-(6-*O*-malonyl)-glucoside, and quercetin-3-*O*-(6-*O*-acetyl)-galactoside) both exhibited significant antimicrobial activity against methicillin-resistant *Staphylococcus aureus*, *Pseudomonas aeruginosa* and the fungus, *Aspergillus flavus*, but *A. pictum* was superior to *A. venetum* in terms of antimicrobial capacity (Gao et al., 2021). Apart from the pharmacological value, in recent years, *A. pictum* is often studied together with *A. venetum* because of its high ecological value.

### The ecological value of *A. venetum* and *A. pictum*

Phytoremediation is one of the appropriate ways to deal with land problems such as drought, salinity and metal pollution (Pilon-Smits, 2005). *Apocynum* spp. were selected to stabilize sands and restore the degraded saline lands due to their advantages of easy propagation, resistance to harsh environment, and high economic value (Jiang et al., 2021a; Jiang et al., 2021b). The matured seeds of *A. venetum* appeared to possess higher drought tolerance than seeds of *A. pictum*. The simulation of the critical values of *Apocynum* spp. seeds under PEG-6000 simulated drought conditions are summarized in Table 5. Different PEG-6000 concentrations (0%–35%) was used to simulate natural drought conditions to study the effect of drought stress on the germination of *Apocynum* spp. seeds. The results showed that low concentrations PEG (0–20%) had no significant impact on the germination rate of *Apocynum* spp. seeds. However, when the concentration was more than 20%, the germination rates of the seeds were reduced, and the negative impact on *A. pictum* seeds was higher than that on *A.venetum*. In addition, after the drought stress was alleviated, the seeds were able to germinate under appropriate conditions (Han et al., 2021; Jiang et al., 2021a). Moreover, the membership function (A mathematical tool for representing fuzzy sets) was used to comprehensively evaluate the drought resistance of *A. venetum* and another desert economic plant, *Lycium ruthenicum,* by analyzing the physiological and biochemical indices (the content of chlorophyll a, chlorophyll b, proline and soluble sugar, antioxidant enzyme activity, *etc*.). The results showed that when the soil moisture content was 9.70%, 6.89% and 5.54%, the drought resistance of *A. venetum* was stronger than that of *Lycium ruthenicum* (Wang, 2017).

**Table 5:** Tolerance value of *Apocynum* spp. under Simulated Drought (PEG) and Salt (NaCl, LiCl) conditions.

Tolerance value	*A. venetum*	*A. pictum*	Reference
Simulated critical value (PEG concentration)	29.56%	26.58%	Jiang et al. (2021a); Jiang et al. (2021b)
Simulated limit value (PEG concentration)	40.16%	39.81%	
Simulated critical value (NaCl concentration)	431 mM	456 mM	
Simulated limit value (NaCl concentration)	653 mM	631 mM	
Simulated critical value (LiCl concentration)	196 mM	235 mM	Jiang, Wang & Tian (2018a); Jiang et al. (2018b)
Simulated limit value (LiCl concentration)	428 mM	406 mM	

Low concentration of salt solution (0–200 mM NaCl) had no significant effect on the germination rate of current season mature seeds the two species (Jiang et al., 2021a; Shi et al., 2014). However, another study showed that under 200 mM NaCl stress, the growth and development of *A. venetum* seedlings were inhibited, the phenotypic characteristics (plant height, root length, leaf length, leaf width) were damaged, and the total flavonoid content decreased. However, salt stress increased the content of quercetin and kaempferol in seedlings (Xu et al., 2020b). In addition, the seeds of *Apocynum* spp. both exhibited high tolerance to lithium salts during germination, particularly LiCl (Table 5) (Gao et al., 2020; Jiang, Wang & Tian, 2018a; Jiang et al., 2018b). The simulated critical value of *A. venetum* was as high as 196 mM (Jiang, Wang & Tian, 2018a). To put the salt tolerance of *A. venetum* into perspective, *Brassica carinata*, another heavy metal tolerant plant with phytoremediation potential, has a germination rate of less than 50% at LiCl concentration above 120 mM (Li et al., 2009). Notably, the addition of lithium in soil did not reduce the concentrations and antioxidant capacity of total flavonoids, rutin and hyperoside in *A. venetum* leaves (Jiang et al., 2019). Therefore, *Apocynum* spp. are suitable for the restoration of degraded saline soil in arid areas, and are promising species in the remediation of lithium pollution in the environment (Jiang et al., 2021a; Jiang et al., 2021b; Rouzi et al., 2018).

## Conclusions

Looking back on the research history of *A. venetum*, the research focuses mainly on the components and pharmacological effects of *A. venetum* leaves. At present, many of the pharmacological effects are attributable to flavonoids, however these active components and their synergistic mechanism need to be further studied. In addition to flavonoids, some polysaccharides (Vp2a-II, Vp3) and triterpenoid (uvaol) from *A. venetum* have also shown pharmacological effects. However, the current research in this area is still lacking. In recent trends, the fiber of *A. venetum* have attracted attention. Apart from its textile value, the potential application of the fiber in other industries needs further exploration in future studies. The ecological value of *Apocynum* spp. is gradually being revealed by multiple research.

This study provided rich and rigorous CiteSpace analysis on *A. venetum*. However, as a limitation, we analyzed only the papers written in English, and within the WoS database, therefore it may not be comprehensive enough to reflect the entire research status. For example, we searched a major Chinese scientific literature database, the China National Knowledge Infrastructure (CNKI), and more than 2,000 *Apocynum* related publications were retrieved, although these were not within the analysis scope of the current study. This further attests to the interest *Apocynum* species have received from the scientific community over the past decades.

##  Supplemental Information

10.7717/peerj.14966/supp-1 Supplemental Information 1 Raw data analyzed by CiteSpace

10.7717/peerj.14966/supp-2 Supplemental Information 2 Documented subspecies of *A. venetum.* Source: World Flora Online, https://wfoplantlist.org/plant-list/taxon/wfo-0000245931-2022-12.

## Additional Information and Declarations

### Data Availability

The following information was supplied regarding data availability: The datasets analyzed are available in the Supplementary File and at the Web of Science Core Collection: https://wfoplantlist.org/plant-list/taxon/wfo-0000245931-2022-12.

## References

[ref-1] Abubakar AS, Gao G, Zhu A (2021). Apocynum venetum, a bast fiber plant with medicinal significances and potentials for drought tolerance and phytoremediation studies—a review. Journal of Natural Fibers.

[ref-2] An H, Wang H, Lan Y, Hashi Y, Chen S (2013). Simultaneous qualitative and quantitative analysis of phenolic acids and flavonoids for the quality control of *Apocynum venetum* L. leaves by HPLC-DAD-ESI-IT-TOF-MS and HPLC-DAD. Journal of Pharmaceutical and Biomedical Analysis.

[ref-3] Chan CO, Lau CC, Ng YF, Xu LJ, Chen SB, Chan SW, Mok DK (2015). Discrimination between Leave of *Apocynum venetum* and its adulterant, a. pictum based on antioxidant assay and chemical profiles combined with multivariate statistical analysis. Antioxidants.

[ref-4] Chen C, Hu Z, Liu S, Tseng H (2012). Emerging trends in regenerative medicine: a scientometric analysis in CiteSpace. Expert Opinion on Biological Therapy.

[ref-5] Chen C, Xu F, Ji Q, Xu D, Yu T, Li Z (2022a). Study on the preparation and properties of Xinjiang *Apocynum Venetum* Fiber. Journal of Natural Fibers.

[ref-6] Chen X, Wang C, Wang C, Liu C, Yuan Y, Wang B, Wu G, Han Y, Zhao Y, Wu Z, Li X (2022b). The emulsification properties of alkaline-extracted polysaccharide conjugates from Apocynum venetum L. tea residues. Food Hydrocolloids.

[ref-7] Chinese Pharmacopoeia C (2020). Pharmacopoeia of the People’s Republic of China.

[ref-8] Du SY, Huang HF, Li XQ, Zhai LX, Zhu QC, Zheng K, Song X, Xu CS, Li CY, Li Y, He ZD, Xiao HT (2020). Anti-inflammatory properties of uvaol on DSS-induced colitis and LPS-stimulated macrophages. Chinese Medicine.

[ref-9] Duan S, Cheng L, Feng X, Zheng K, Peng Y, Liu Z (2017). Bio-degumming technology of *Apocynum venetum* bast by *Pectobacterium* sp. DCE-01. Textitle Research Journal.

[ref-10] Duan S, Xu B, Cheng L, Feng X, Yang Q, Zheng K, Gao M, Liu Z, Liu C, Peng Y (2021). Bacterial strain for bast fiber crops degumming and its bio-degumming technique. Bioprocess and Biosystems Engineering.

[ref-11] Fu H-M, Yin C-L, Shen Z-Y, Yang M-H (2022). Flavonoids from the leaves of Apocynum venetum and their anti-inflammatory activity. Journal of Chemical Research.

[ref-12] Gao G, Chen P, Chen J, Chen K, Wang X, Abubakar AS, Liu N, Yu C, Zhu A (2019). Genomic survey, transcriptome, and metabolome analysis of *Apocynum venetum* and *Apocynum hendersonii* to reveal major flavonoid biosynthesis pathways. Metabolites.

[ref-13] Gao G, Hazaisi H, Yu C, Chen P, Chen J, Chen K, Liu N, Zhu A (2020). Effects of LiCl stress on seed germination and subcellular distribution of Li ^+^ in *Apocynum venetum* and *Apocynum hendersonii* (Hook.f.). Plant Fiber Sciences in China.

[ref-14] Gao G, Liu N, Yu C, Chen P, Chen J, Chen K, Wang X, Liu B, Zhu A (2021). UPLC-ESI-MS/MS based characterization of active flavonoids from *apocynum* spp. and anti-bacteria assay. Antioxidants.

[ref-15] Grundmann O, Nakajima J, Seo S, Butterweck V (2007). Anti-anxiety effects of *Apocynum venetum* L. in the elevated plus maze test. Journal of Ethnopharmacology.

[ref-16] Guo H, Kuang Z, Zhang J, Zhao X, Pu P, Yan J (2020). The preventive effect of *Apocynum venetum* polyphenols on D-galactose-induced oxidative stress in mice. Experimental and Therapeutic Medicine.

[ref-17] Halim AF, Lv Z, Yida C, Mingbo M, Liu H, Zhou W (2020). Fidelity of new chemical degumming method for obtaining superior properties of Bast fiber from *Apocynum venetum*. Textile Research Journal.

[ref-18] Halim A (2021). Extraction and characterization of microcrystalline cellulose from *Apocynum venetum*.

[ref-19] Han F-G, Xu X-Y, Ma Q-L, Man D-Q, Zheng Q-Z, Wei L-Y (2021). Response of seed germination of *Poacynum hendersonii* and *Apocynum venetum* to drought stress. Journal of Northwest Forestry University.

[ref-20] Han G, Wang L, Liu M, Zhang Y (2008). Component analysis and microfiber arrangement of *Apocynum venetum* fibers: the MS and AFM study. Carbohydrate Polymers.

[ref-21] Hao XL, Kang Y, Li JK, Li QS, Liu EL, Liu XX (2016). Protective effects of hyperoside against H2O2-induced apoptosis in human umbilical vein endothelial cells. Molecular Medicine Reports.

[ref-22] Huang SP, Ho TM, Yang CW, Chang YJ, Chen JF, Shaw NS, Horng JC, Hsu SL, Liao MY, Wu LC, Ho JA (2017). Chemopreventive potential of ethanolic extracts of luobuma leaves (*Apocynum venetum* L.) in androgen insensitive prostate cancer. Nutrients.

[ref-23] Jiang L, She C, Tian C, Tanveer M, Wang L (2021a). Storage period and different abiotic factors regulate seed germination of two apocynum species - cash crops in arid saline regions in the Northwestern China. Frontiers in Plant Science.

[ref-24] Jiang L, Wang L, Tanveer M, Tian C (2019). Lithium biofortification of medicinal tea *Apocynum venetum*. Scientific Reports.

[ref-25] Jiang L, Wang L, Tian CY (2018a). High lithium tolerance of *Apocynum venetum* seeds during germination. Environmental Science and Pollution Research.

[ref-26] Jiang L, Wang L, Zhang L, Tian C (2018b). Tolerance and accumulation of lithium in Apocynum pictum Schrenk. PeerJ.

[ref-27] Jiang L, Wu X, Zhao Z, Zhang K, Tanveer M, Wang L, Huang J, Tian C, Wang L (2021b). Luobuma (Apocynum) –cash crops for saline lands. Industrial Crops and Products.

[ref-28] Jin Y, Yang Wang C, Hu W, Huang Y, Li Xu M, Wang H, Kong X, Chen Y, Dong TT, Qin Q, Keung Tsim KW (2019). An optimization of ultra-sonication-assisted extraction from flowers of Apocynum venetum in targeting to amount of free amino acids determined by UPLC-MS/MS. Food Quality and Safety.

[ref-29] Kong NN, Fang ST, Liu Y, Wang JH, Yang CY, Xia CH (2014). Flavonoids from the halophyte *Apocynum venetum* and their antifouling activities against marine biofilm-derived bacteria. Natural Product Research.

[ref-30] Lau YS, Kwan CY, Ku TC, Hsieh WT, Wang HD, Nishibe S, Dharmani M, Mustafa MR (2012). Apocynum venetum leaf extract, an antihypertensive herb, inhibits rat aortic contraction induced by angiotensin II: a nitric oxide and superoxide connection. Journal of Ethnopharmacology.

[ref-31] Li C, Liu S, Song Y, Nie K, Ben H, Zhang Y, Han G, Jiang W (2020). A facile and eco-friendly method to extract *Apocynum venetum* fibers using microwave-assisted ultrasonic degumming. Industrial Crops and Products.

[ref-32] Li M, Han G, Chen H, Yu J, Zhang Y (2012). Chemical compounds and antimicrobial activity of volatile oils from bast and fibers of Apocynum venetum. Fibers and Polymers.

[ref-33] Li X, Gao P, Gjetvaj B, Westcott N, Gruber MY (2009). Analysis of the metabolome and transcriptome of Brassica carinata seedlings after lithium chloride exposure. Plant Science.

[ref-34] Liu D, Wang S-Y, Bao Y-L, Zheng L-H, Wang G-N, Sun Y, Yang X-G, Liu L (2022). Extraction, purification and structural characterization of polysaccharides from *Apocynum venetum* L. roots with anti-inflammatory activity. Process Biochemistry.

[ref-35] Liu J, Song Y, Han G, Han Y, Zhang Y, Jiang W (2020). The dimensional distribution of kenaf and apocynum fibers. Journal of Natural Fibers.

[ref-36] Lou J, Yao L, Qiu Y, Lin H, Kuang Y, Qi S (2019). The chemical degumming process and effect on the composition, structure and properties of *Apocynum venetum*. Textile Research Journal.

[ref-37] Manzoor M, Muroi M, Ogawa N, Kobayashi H, Nishimura H, Chen D, Fasina OB, Wang J, Osada H, Yoshida M, Xiang L, Qi J (2022). Isoquercitrin from *Apocynum venetum* L. produces an anti-obesity effect on obese mice by targeting C-1-tetrahydrofolate synthase, carbonyl reductase, and glutathione S-transferase P and modification of the AMPK/SREBP-1c/FAS/CD36 signaling pathway in mice in vivo. Food & Function.

[ref-38] National Health Commission of the People’s Republic China (2002). Notice of the Ministry of health on further standardizing the management of health food raw materials. http://www.nhc.gov.cn/sps/s3593/200810/bc239ea3d226449b86379f645dfd881d.shtml.

[ref-39] Pilon-Smits E (2005). Phytoremediation. Annual Review of Plant Biology.

[ref-40] Rouzi A, Halik Ü, Thevs N, Welp M, Aishan T (2018). Water efficient alternative crops for sustainable agriculture along the Tarim basin: a comparison of the economic potentials of *Apocynum pictum*, Chinese red date and cotton in Xinjiang, China. Sustainability.

[ref-41] Shi Q, Deng F, Wu M, Chen D, Yin C (2014). Study on Salt Tolerance of *Apocynum venetum* Linn. and *Poacynum hendersonii* (Hook.f.) Woodson at stages of seed germination and seedings growth. Northern Horticulture.

[ref-42] Song Y, Kai N, Jiang W, Zhang Y, Ben H, Han G, Ragauskas AJ (2019). Utilization of deep eutectic solvent as a degumming protocol for *Apocynum venetum* bast. Cellulose.

[ref-43] Sun S, Zhao Y, Wang L, Tan Y, Shi Y, Sedjoah R-CA-A, Shao Y, Li L, Wang M, Wan J, Fan X, Guo R, Xin Z (2022). Ultrasound-assisted extraction of bound phenolic compounds from the residue of *Apocynum venetum* tea and their antioxidant activities. Food Bioscience.

[ref-44] Thevs N, Zerbe S, Kyosev Y, Rouzi A, Tang B, Abdusalih N, Novitskiy Z (2012). Apocynum venetum L. and *Apocynum pictum* Schrenk (Apocynaceae) as multi-functional and multi-service plant species in Central Asia: a review on biology, ecology, and utilization. Journal of Applied Botany and Food Quality.

[ref-45] Wang Y, Berhow MA, Black M, Jeffery EH (2020). A comparison of the absorption and metabolism of the major quercetin in brassica, quercetin-3-O-sophoroside, to that of quercetin aglycone, in rats. Food Chemistry.

[ref-46] Wang C, Wang L, Zhang Q, Cheng L, Yue H, Xia X, Zhou H (2021a). Preparation and characterization of *Apoacynum venetum* cellulose nanofibers reinforced chitosan-based composite hydrogels. Colloids and Surfaces B: Biointerfaces.

[ref-47] Wang H, Ma C, Sun-Waterhouse D, Wang J, Waterhouse GINeil, Kang W (2022). Immunoregulatory polysaccharides from *Apocynum venetum* L. flowers stimulate phagocytosis and cytokine expression via activating the NF-*κ*B/MAPK signaling pathways in RAW264.7 cells. Food Science and Human Wellness.

[ref-48] Wang J (2017). Study on drought resistance of two species of economic desert plant of *Lycium ruthenicum* and *Apocynum venetum* Master.

[ref-49] Wang L, Han G, Zhang Y (2007). Comparative study of composition, structure and properties of *Apocynum venetum* fibers under different pretreatments. Carbohydrate Polymers.

[ref-50] Wang L, Wang C, Wang L, Zhang Q, Wang Y, Xia X (2021b). Emulsion electrospun polylactic acid/Apocynum venetum nanocellulose nanofiber membranes with controlled sea buckthorn extract release as a drug delivery system. Textile Research Journal.

[ref-51] Wang L, Wang C, Zhang Q, Liu J, Xia X (2019a). Comparison of morphological, structural and antibacterial properties of different *Apocynum venetum* poly (lactic acid)/nanocellulose nanofiber films. Textile Research Journal.

[ref-52] Wang L, Zhang X, Niu Y, Ahmed AF, Wang J, Kang W (2019b). Anticoagulant activity of two novel polysaccharides from flowers of *Apocynum venetum* L. International Journal of Biological Macromolecules.

[ref-53] World Flora Online (2022). https://wfoplantlist.org/plant-list/taxon/wfo-0000245931-2022-12.

[ref-54] Wu R, Yakhkeshi S, Zhang X (2022). Scientometric analysis and perspective of IgY technology study. Poultry Science.

[ref-55] Xie W, Chen C, Jiang Z, Wang J, Melzig MF, Zhang X (2015). *Apocynum venetum* attenuates acetaminophen-induced liver injury in mice. The American Journal of Chinese Medicine.

[ref-56] Xie W, Jiang Z, Wang J, Zhang X, Melzig MF (2016a). Protective effect of hyperoside against acetaminophen (APAP) induced liver injury through enhancement of APAP clearance. Chemico-Biological Interactions.

[ref-57] Xie W, Wang M, Chen C, Zhang X, Melzig MF (2016b). Hepatoprotective effect of isoquercitrin against acetaminophen-induced liver injury. Life Sciences.

[ref-58] Xie W, Zhang X, Wang T, Hu J (2012). Botany, traditional uses, phytochemistry and pharmacology of *Apocynum venetum* L. (Luobuma): a review. Journal of Ethnopharmacology.

[ref-59] Xu X, Gong J, Zhang T, Li Z, Zhang J, Wang L, Huang J (2020a). Insights into antibacterial mechanism of *Apocynum Venetum* L. fiber: evolution of bioactive natural substances in bast during chemical degumming process. Industrial Crops and Products.

[ref-60] Xu Z, Zhou J, Ren T, Du H, Liu H, Li Y, Zhang C (2020b). Salt stress decreases seedling growth and development but increases quercetin and kaempferol content in *Apocynum venetum*. Plant Biology.

[ref-61] Yan S-X, Lang J-L, Song Y-Y, Wu Y-Z, Lv M-H, Zhao X, Liu Y-H, Xu C-Y (2016). Studies on anti-depressant activity of four flavonoids isolated from *apocynum venetum* Linn (Apocynaceae) leaf in mice. Tropical Journal of Pharmaceutical Research.

[ref-62] Yang F, Ma Y, Qian Y, Lv L, Zheng L, Zhao Y (2015). A novel ionic liquid degumming process for *Apocynum venetum*. The Journal of The Textile Institute.

[ref-63] Yang F, Ma Y, Zheng H, Zheng L, Zhao Y (2019). An eco-friendly Degumming of Apocynum Venetum with A ionic liquid pretreatment. Journal of Natural Fibers.

[ref-64] Yuan N, Li M, Jia C (2020a). De novo transcriptome assembly and population genetic analyses of an important coastal shrub, *Apocynum venetum* L. BMC Plant Biology.

[ref-65] Yuan Y, Zhou J, Zheng Y, Xu Z, Li Y, Zhou S, Zhang C (2020b). Beneficial effects of polysaccharide-rich extracts from *Apocynum venetum* leaves on hypoglycemic and gut microbiota in type 2 diabetic mice. Biomed Pharmacother.

[ref-66] Zeng S, Zhao X, Xu LS, Yang D, Chen L, Xu MH (2019). Apoptosis induction effect of *Apocynum venetum* polyphenol on human U87 glioma cells via NF-*κ*B pathway. Future Oncology.

[ref-67] Zhao L, Liang S, Lv L, Zhang H, Guo-Tan G, Chai Y, Zhang G (2014). Screening and analysis of metabolites in rat urine after oral administration of *Apocynum venetum* L. extracts using HPLC-TOF-MS. Journal of Separation Science.

[ref-68] Zhang L, Yu ZY, Wang H, Jiang L, Zhan YG, Fan GZ (2021). Flavonoid production and antioxidative activity in liquid-cultured hairy roots of *Apocynum venetum*. Journal of Plant Biochemistry and Biotechnology.

[ref-69] Zhang Y, Liu S, Ma JL, Chen C, Huang P, Ji JH, Wu D, Ren LQ (2022). Apocynum venetum leaf extract alleviated doxorubicin-induced cardiotoxicity through the AKT/Bcl-2 signaling pathway. Phytomedicine.

[ref-70] Zheng C, Fan J, Caraballo-Ortiz MA, Liu Y, Liu T, Fu G, Zhang Y, Yang P, Su X (2022). The complete chloroplast genome and phylogenetic relationship of *Apocynum pictum* (Apocynaceae), a Central Asian shrub and second-class national protected species of western China. Gene.

[ref-71] Zheng M, Liu C, Pan F, Shi D, Zhang Y (2012). Antidepressant-like effect of hyperoside isolated from *Apocynum venetum* leaves: possible cellular mechanisms. Phytomedicine.

[ref-72] Zhou J, Zou P, Jing C, Xu Z, Zhou S, Li Y, Zhang C, Yuan Y (2019). Chemical characterization and bioactivities of polysaccharides from *Apocynum venetum* leaves extracted by different solvents. Journal of Food Measurement and Characterization.

