# Supplementary material for: Apocynum venetum, a medicinal, economical and ecological plant: a review update
Source: PeerJ. 2023 Mar 7;11:e14966. doi: 10.7717/peerj.14966 (PMC10000306; doi:10.7717/peerj.14966)
Supplement: Supplemental Information 2 — Source: World Flora Online, https://wfoplantlist.org/plant-list/taxon/wfo-0000245931-2022-12. [file peerj-11-14966-s002.docx]

Table S1: Documented subspecies of *A. venetum.*

| Subspecies | Synonyms | Native range |
| --- | --- | --- |
| *Apocynum venetum subsp. armenum* (Pobed.) ined. | *Apocynum armenum* Pobed.  *Poacynum armenum* (Pobed.) Mavrodiev, Laktionov & Yu.E.Alexeev  *Trachomitum armenum* (Pobed.) Pobed.  *Trachomitum venetum subsp. armenum* (Pobed.) Rech.f. | Eastern. Türkiye to Iran |
| *Apocynum venetum subsp. basikurumon* (H.Hara) ined. | *Apocynum basikurumon* Hara | Japan |
| *Apocynum venetum subsp. lancifolium* (Russanov) ined. | *Apocynum compressum* Moench;  *Nerium antidysentericum* Lepech.  *Nerium sibiricum* Medik.  *Poacynum lancifolium* (Russanov) Mavrodiev, Laktionov & Yu.E.Alexeev  *Trachomitum lancifolium* (Russanov) Pobed. | Southwest. & South. Siberia to Korea |
| *Apocynum venetum subsp. russanovii* (Pobed.) ined. | *Apocynum russanovii* Pobed.  *Poacynum russanovii* (Pobed.) Mavrodiev, Laktionov & Yu.E.Alexeev  *Trachomitum russanovii* (Pobed.) Pobed.  *Trachomitum venetum subsp. russanovii* (Pobed.) Yena & Moysienko | South. Ukraine (Ostriv Dzharylhach). |
| *Apocynum venetum subsp. sarmatiense* (Woodson) ined. | *Apocynum sarmatiense* (Woodson) Wissjul.  *Poacynum kazakevichii* Mavrodiev, Laktionov & Yu.E.Alexeev  *Poacynum sarmatiense* (Woodson) Mavrodiev, Laktionov & Yu.E.Alexeev  *Trachomitum sarmatiense* Woodson  *Trachomitum venetum subsp. sarmatiense* V.E.Avet. | Eastern. & Southeast. Europe to Iran. |
| *Apocynum venetum subsp. scabrum* (Russanov) ined. | *Poacynum scabrum* (Russanov) Mavrodiev, Laktionov & Yu.E.Alexeev  *Trachomitum scabrum* (Russanov) Pobed.  *Trachomitum venetum subsp. scabrum* (Russan.) Rech.f.  *Trachomitum venetum var. scabrum* Kitam. | Iran to Central Asia and Pakistan |
| *Apocynum venetum subsp. tauricum* (Pobed.) ined. | *Apocynum tauricum* Pobed.  *Poacynum tauricum* (Pobed.) Mavrodiev, Laktionov & Yu.E.Alexeev  *Trachomitum tauricum* (Pobed.) Pobed.  *Trachomitum venetum subsp. tauricum* (Pobed.) Greuter & Burdet | Southeast. Krym (Cape St. Ilya) |
| *Apocynum venetum subsp. venetum* | *Apocynum venetum var. ellipticifolium* Bég. & Belosersky  *Asclepias rubra* Siev.  *Poacynum ellipticifolium* (Bég. & Belosersky) Mavrodiev, Laktionov & Yu.E.Alexeev | Italy |
| *Apocynum venetum var. scabrum* (Bég. & Bél.) Bég. & Bél. |  | Iran to Central Asia and Pakistan. |

Note: the information was sources from World Flora Online (<https://wfoplantlist.org/plant-list/taxon/wfo-0000245931-2022-12>).
